# Neuraxial anesthesia for orthopedic surgery: systematic review and meta-analysis of randomized clinical trials

**DOI:** 10.1590/1516-3180.2013.1316667

**Published:** 2013-12-01

**Authors:** Fabiano Timbó Barbosa, Aldemar Araújo Castro, Célio Fernando de Sousa-Rodrigues

**Affiliations:** I MSc. Professor, Department of Anesthesiology, Universidade Federal de Alagoas (UFA), Maceió, Alagoas, Brazil.; II MSc. Assistant Professor, Department of Surgery, Universidade Estadual de Ciências da Saúde de Alagoas (UNCISAL), Maceió, Alagoas, Brazil.; III PhD. Adjunct Professor, Department of Anatomy, Universidade Estadual de Ciências da Saúde de Alagoas (UNCISAL), Maceió, Alagoas, Brazil.

**Keywords:** Mortality, Anesthesia, general, Anesthesia, epidural, Anesthesia, spinal, Review [publication type], Mortalidade, Anestesia geral, Anestesia epidural, Raquianestesia, Revisão

## Abstract

**CONTEXT AND OBJECTIVE:**

Taking the outcome of mortality into consideration, there is controversy about the beneficial effects of neuraxial anesthesia for orthopedic surgery. The aim of this study was to compare the effectiveness and safety of neuraxial anesthesia versus general anesthesia for orthopedic surgery.

**DESIGN AND SETTING:**

Systematic review at Universidade Federal de Alagoas.

**METHODS:**

We searched the Cochrane Central Register of Controlled Trials (Issue 10, 2012), PubMed (1966 to November 2012), Lilacs (1982 to November 2012), SciELO, EMBASE (1974 to November 2012) and reference lists of the studies included. Only randomized controlled trials were included.

**RESULTS:**

Out of 5,032 titles and abstracts, 17 studies were included. There were no statistically significant differences in mortality (risk difference, RD: -0.01; 95% confidence interval, CI: -0.04 to 0.01; n = 1903), stroke (RD: 0.02; 95% CI: -0.04 to 0.08; n = 259), myocardial infarction (RD: -0.01; 95% CI: -0.04 to 0.02; n = 291), length of hospitalization (mean difference, -0.05; 95% CI: -0.69 to 0.58; n = 870), postoperative cognitive dysfunction (RD: 0.00; 95% CI: -0.04 to 0.05; n = 479) or pneumonia (odds ratio, 0.61; 95% CI: 0.25 to 1.49; n = 167).

**CONCLUSION:**

So far, the evidence available from the studies included is insufficient to prove that neuraxial anesthesia is more effective and safer than general anesthesia for orthopedic surgery. However, this systematic review does not rule out clinically important differences with regard to mortality, stroke, myocardial infarction, length of hospitalization, postoperative cognitive dysfunction or pneumonia.

## Abstract (pt)

**CONTEXTO E OBJETIVO:**

Considerando o desfecho de mortalidade, existe controvérsia acerca dos efeitos benéficos da anestesia neuroaxial (AN) para cirurgias ortopédicas. O objetivo do estudo foi comparar efetividade e segurança da AN *versus* anestesia geral (AG) para cirurgias ortopédicas.

**TIPO DE ESTUDO E LOCAL:**

Revisão sistemática na Universidade Federal de Alagoas.

**MÉTODOS:**

Buscamos em Cochrane Central Register of Controlled Trials (2012, volume 10), PubMed (1966 até novembro de 2012), Lilacs (1982 até novembro de 2012), SciELO, EMBASE (1974 até novembro de 2012) e listas de referências dos estudos incluídos. Apenas ensaios clínicos randomizados foram incluídos. RESULTADOS: Dentre 5.032 títulos e resumos, 17 estudos foram incluídos. Não houve diferença estatística em mortalidade (diferença de risco, DR: -0,01; intervalo de confiança de 95%, IC: -0,04 a 0.01; n = 1903), em acidente vascular encefálico (DR: 0,02; IC 95%: -0,04 a 0,08; n = 259, em infarto miocárdico (DR: -0.01; IC 95%: -0,04 a 0.02; n = 291), tempo de hospitalização (diferença média, -0,05; IC 95%: -0,69 a 0,58; n = 870), em disfunção cognitiva pós-operatória (DR: 0,00; IC 95%: -0,04 a 0,05; n = 479) e pneumonia (razão de chances, 0,61; IC 95%: 0,25 a 1,49; n = 167).

**CONCLUSÃO:**

Até o momento, as evidências são insuficientes nos estudos incluídos para provar que AN é mais efetiva e segura do que AG para cirurgias ortopédicas. Esta revisão sistemática não descartou diferenças clínicas importantes para mortalidade, acidente vascular encefálico, infarto miocárdico, tempo de internação, disfunção cognitiva pós-operatória e pneumonia.

## INTRODUCTION

Neuraxial anesthesia combined with postoperative epidural analgesia can reduce the physiological stress attributed to surgery and the incidence of postoperative complications.^1^ There are some advantages to neuraxial anesthesia, such as reductions in the incidence of deep-vein thrombosis and pulmonary embolism, and in the need for blood transfusion.^2^ Although there are advantages, there is some potential for neurological damage and a great degree of hypotension, which may make it less acceptable.^1^ Rodgers et al. published a systematic review demonstrating that neuraxial anesthesia can decrease mortality over the course of the follow-up time, compared with general anesthesia, but these results cannot be used in orthopedic surgery because most of the procedures involved consisted of abdominal surgery.^3^

Neuraxial anesthesia is used routinely in orthopedic surgery, but there is controversy regarding the beneficial effects of this anesthetic technique, taking into consideration the length of follow-up and its correlation with mortality and postoperative morbidity.^4^

Although some systematic reviews have analyzed neuraxial anesthesia for orthopedic surgery, the reviewers looked for the magnitude of the effect in a separated manner, without pooling knee, hip, femur and ankle results in a meta-analysis. Thus, controversy remains regarding the effectiveness and safety of neuraxial anesthesia.^2^ ^,^ ^5^ ^-^ ^8^ In this context, we proposed to answer the research question: what is the effectiveness and safety of neuraxial anesthesia, in comparison with general anesthesia for orthopedic surgery?

## OBJECTIVE

The purpose of this systematic review was to compare the effectiveness and safety of neuraxial anesthesia versus general anesthesia for orthopedic surgery.

## METHODS

### Protocol

A protocol was developed *a priori* and is available from the corresponding author if it needs to be analyzed. This research was conducted in accordance with the Cochrane Collaboration Handbook.^9^ The journals, institutions where the studies were conducted or researchers did not influence our results. The Preferred Reporting Items for Systematic Reviews and Meta-Analyses (PRISMA) statement was followed in relation to reporting items for systematic reviews and meta-analyses.^10^

### Eligibility criteria

*Types of participants:* The patients included in this review were 18 years old or over. They presented orthopedic disorders below the umbilical scar, and were treated surgically. Patients who underwent orthopedic surgery performed together with other types of surgery were excluded. 

*Types of studies:* Randomized controlled trials (RCTs) were included. Data from studies published twice were collected from the original article with the best description. Studies with incomplete outcome data description were excluded from the meta-analysis.

*Types of interventions:* the intervention group was neuraxial anesthesia. The control group was general anesthesia. Use of a catheter in neuraxial anesthesia techniques was not an exclusion criterion.

*Identification of studies*

The following databases were searched: Cochrane Central Register of Controlled Trials (CENTRAL) in the Cochrane Library (Issue 10, 2012); Medline (Medical Analysis and Retrieval System Online) via PubMed (1966 to November 2012); Lilacs (Literatura Latino-Americana e do Caribe em Ciências da Saúde), available at http://regional.bvsalud.org/php/index.php (1982 to November 2012); SciELO (Scientific Electronic Library Online), available at http://www.scielo.br (the last search was in November 2012); and EMBASE (Excerpta Medica database), which is available at http://aplicacao.periodicos.saude.gov.br/ (1974 to November 2012). In addition, the reference lists of the studies included were also searched. There were no restrictions on any language, date and document format. The search strategies used in Medline via PubMed were adapted and used for CENTRAL. We used the terms anesthesia and orthopedic surgeries for Lilacs. We used the terms anesthesia and urology for SciELO. The search strategy for EMBASE was 'general anesthesia'/exp OR 'spinal anesthesia'/exp OR 'epidural anesthesia'/exp AND rand* AND 'orthopedic surgery'/exp [embase]/lim. The search strategy for PubMed can be seen in Table 1.

**Table 1:** Search strategies for Medline via PubMed

Database	Search strategy
PubMed	(Therapy/Broad[filter])
	AND
	("anesthesia, general"[MeSH Terms] OR "anesthesia, inhalation"[MeSH Terms] OR "anesthesia, intravenous"[MeSH Terms] AND "anesthesia, conduction"[MeSH Terms] OR "anesthesia, epidural"[MeSH Terms] OR "anesthesia, spinal" [MeSH Terms])
	AND
	"orthopedics"[MeSH Terms] OR Orthopedics [Text Word])

*Selection of studies*

Titles, abstracts, or both, identified through the search strategy for all databases, were independently reviewed by two investigators (Barbosa FT and Rodrigues CFS). RCTs identified in accordance with our eligibility criteria were obtained in order to read the full text. A standardized form was developed by the authors and was used to collect data. Discordances were resolved through consensus meetings.

### Methodological quality and risk-of-bias assessment

The study validity of the RCTs was independently assessed by two authors (Barbosa FT and Rodrigues CFS). Discordances were resolved through consensus meetings. The risk of bias was determined by means of the Rob table, as recommended by the Cochrane Handbook.^9^ The Rob table analyzes sequence generation, allocation sequence concealment, blinding, incomplete outcome data, selective outcome reporting and other sources of bias. Each item was judged subjectively, looking for bias. Three answers were possible: low risk of bias, high risk of bias, or unclear risk of bias. This instrument generated a figure showing the risk-of-bias summary for each study included. 

### Outcomes

The primary outcome was mortality. Mortality was defined as a fatal event during surgery or occurring within one year afterwards.^3^

The secondary outcomes were stroke, myocardial infarction, length of hospitalization, quality of life, degree of satisfaction, postoperative cognitive dysfunction, blood transfusion requirements and pneumonia. We considered stroke to be a loss of brain function caused by a disturbance in brain blood supply. Myocardial infarction was considered to be a loss of cardiac function caused by a disturbance in coronary blood supply. Length of hospitalization was the duration of hospital stay. Quality of life was the aspect of life that was influenced by physical wellbeing or mental status.^9^ Degree of satisfaction was the patient's reaction to the healthcare received.^11^ Postoperative cognitive dysfunction was a state of mental confusion after orthopedic surgery. Blood transfusion requirement was considered to be the number of blood units transfused. Pneumonia was lung infection with changes in pulmonary radiography that started at least 48 hours after surgery.

### Data analysis

For dichotomous outcomes, the odds ratio and 95% confidence interval (CI) were calculated using a random-effect model (REM). When the effect was absent, the risk difference (RD) and 95% confidence interval were calculated using REM. For continuous outcomes, the mean and standard deviation were used to generate the mean difference (MD) and 95% CI using REM. The Rev Man 5 statistical package (Cochrane Collaboration) was used to perform meta-analyses.^12^ I^2^ statistical heterogeneity was assessed by using heterogeneity tests, i.e. the standard chi-square test (P-value < 0.10 or < 10%) and the I^2^ test (I^2^ > 50% was statistically significant). 

## RESULTS

### Study selection

**Figure 1:**
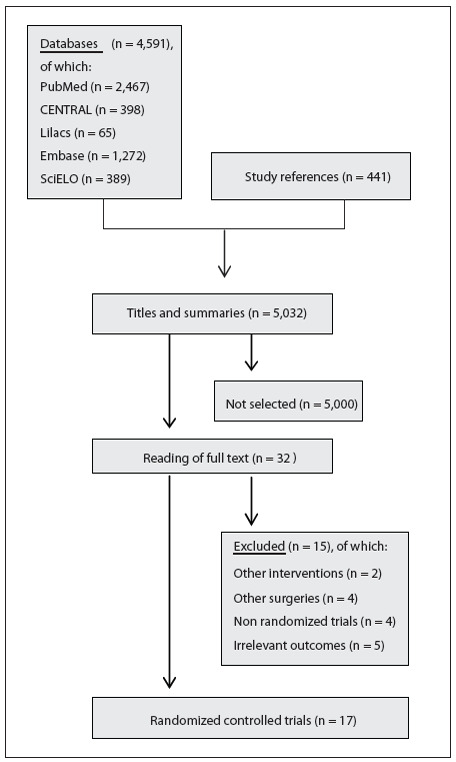
Flow diagram summarizing the process for selecting original articles.

A flow diagram demonstrating the process for selecting relevant articles is outlined in Figure 1. In total, 5032 titles and abstracts were screened. We analyzed 4591 titles after running the search strategy, and 32 papers were identified as relevant through this process.^13^ ^-^ ^44^ Fifteen of these were subsequently excluded.^13^ ^,^ ^14^ ^,^ ^17^ ^,^ ^19^ ^,^ ^20^ ^,^ ^21^ ^,^ ^24^ ^,^ ^26^ ^,^ ^28^ ^,^ ^31^ ^-^ ^33^ ^,^ ^36^ ^,^ ^40^ ^,^ ^43^ The reasons for their exclusion can be seen in Figure 1. Thus, 17 articles with the potential to answer our research question were identified.^15^ ^,^ ^16^ ^,^ ^18^ ^,^ ^22^ ^,^ ^23^ ^,^ ^25^ ^,^ ^27^ ^,^ ^29^ ^,^ ^30^ ^,^ ^34^ ^,^ ^35^ ^,^ ^37^ ^-^ ^39^ ^,^ ^41^ ^,^ ^42^ ^,^ ^44^ We also analyzed 441 titles from the reference lists of these 17 studies included, but did not find any additional studies (Figure 1).

### Study validity

**Figure 2:**
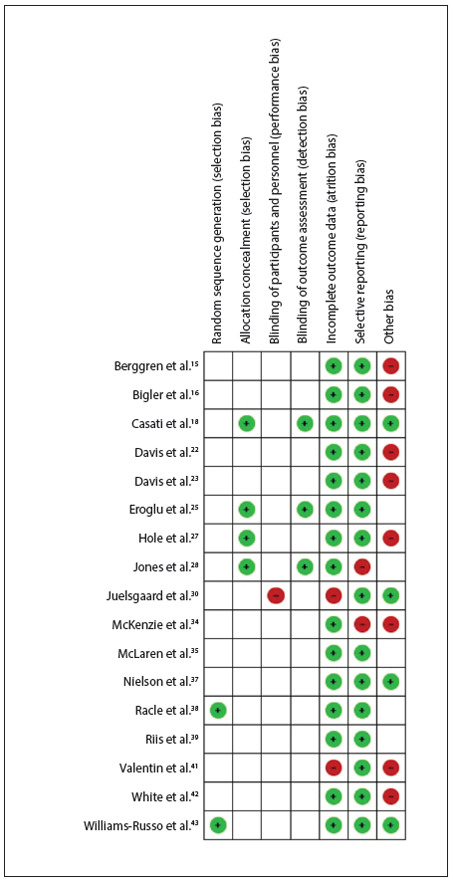
Methodological quality summary: risk of bias for each study included.

The studies were evaluated in accordance with the Rob table from the Cochrane Collaboration,^9^ and were classified as presenting moderate risk of bias because the sequence generation, allocation concealment and blinding method were considered unclear, as presented in Figures 2 ^15^ ^,^ ^18^ ^,^ ^22^ ^,^ ^23^ ^,^ ^25^ ^,^ ^27^ ^,^ ^28^ ^,^ ^30^ ^,^ ^34^ ^,^ ^35^ ^,^ ^37^ ^-^ ^39^ ^,^ ^41^ ^-^ ^43^ and 3.^22^ ^,^ ^23^ ^,^ ^27^ ^,^ ^30^ ^,^ ^34^ ^,^ ^35^ ^,^ ^38^ ^,^ ^41^ ^-^ ^43^ There were some attempts to contact the authors to clarify doubts, but no reply was obtained from them.

**Figure 3:**
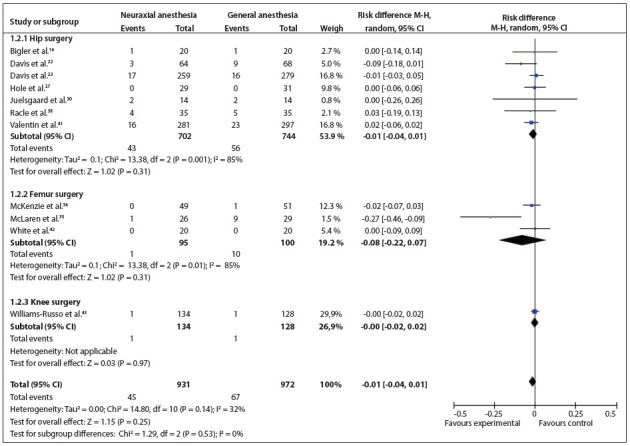
Forest plot including the eleven studies that analyzed mortality.

*Sequence generation.* Two studies included in this systematic review were considered to have low risk of bias.^38^ ^,^ ^44^ The other studies did not present the method used to generate the allocation sequence.

*Allocation concealment.* The method used by the authors to conceal the allocation sequence was described correctly in four of the studies included.^18^ ^,^ ^25^ ^,^ ^27^ ^,^ ^29^ The other studies did not report any details regarding allocation concealment that would make it possible to determine whether this process had been free of bias.

*Blinding of participants, personnel and outcome assessors.* One study was considered to present high risk of bias because although the authors reported that the investigator was blinded to randomization, they did not describe the condition of the participants and the outcome assessors.^30^ The other studies included did not report sufficient details to determine whether they presented high or low risk of bias, or they reported that the outcome assessors were blinded.

*Incomplete outcome data.* In the study by Valentin et al., the result relating to blood loss was reported in a figure, but this presentation did not allow the numbers to be seen and readers were left to estimate the amount of blood loss.^41^ In the study by Juelsgaard et al., usable Holter data were obtained from 43 patients out of the 54 participants.^30^

*Selective reporting.* In two of the studies included, the authors reported results relating to outcomes that were not described in the method.^29^ ^,^ ^34^

*Other sources of bias.* Eight studies received the classification of high risk of bias. In the study by Berggren et al., a scale used to analyze postoperative confusion was modified by the authors and the validation process for this new scale was not tested.^15^ In the study by Bigler et al., the t test was used in the data analysis, but these data presented asymmetric distribution.^16^ In the study by Davis et al., the participants may not have received the same intervention, because the anesthesia was administered by "the duty registrar or consultant anesthetist".^22^ In another study by Davis et al., the follow-up time may not have been the same for all participants.^23^ In the study by Hole et al., patients of different ages received different premedication, and the surgical technique may not have been the same because thirteen surgeons participated in this study.^27^ In the study by McKenzie et al., statistical tests and significance level were not described.^34^ In the study by Valentin et al., there were more ill patients in one group.^41^ In the study by White et al., the authors reported that special attention was given to any respiratory problems during the postoperative period, but they did not report what this attention comprised.^42^

### Outcomes

The characteristics of the selected RCTs analyzed and their outcomes are shown in Table 2.15,16,18,22,23,25,27,29,30,34,35,37-39,41,42,44 It was not possible to pool the data for degree of satisfaction, blood transfusion requirements or quality of life. The data on the degree of satisfaction and blood transfusion requirements were not described correctly in the studies included. The authors did not report any data about quality of life in the studies included.

**Table 2:** Characteristics of the randomized controlled trials that compared the kinds of anesthesia for orthopedic surgery and their contribution to the meta-analysis

Study (year)	Anesthesia	n	Surgery	Outcomes studied	Remark
Berggren et al.15	GA	29	Femoral neck fracture surgery	Stroke, cognitive dysfunction, pneumonia	Only fully lucid patients were included in the study
	EA	28			
Bigler et al.16	GA	20	Hip surgery	Mortality, cognitive dysfunction	Patients with severe dementia, cancer and psychiatric or disseminated neurological disease were not studied
	SA	20			
Casati et al.18	GA	15	Hemiarthroplasty of the hip	Length of hospitalization, cognitive dysfunction	One woman in each group
	USA	15			
Davis et al.22	GA	68	Emergency hip surgery	Mortality, stroke, myocardial infarction	General anesthetic technique may not have been the same for all patients
	SP	64			
Davis et al.23	GA	279	Hip fracture surgery	Mortality, length of hospitalization	Multicenter study; the length of follow-up was not the same for all patients
	SA	259			
Eroglu et al.25	GA	20	Total hip replacement	Blood transfusion requirements	Hypotensive anesthesia was used in both groups
	EA	20			
Hole et al.27	GA	31	Total hip arthroplasty	Mortality, myocardial infarction, degree of satisfaction	Only changes in mental status were analyzed, such as amnesia regarding personal data and disorientation relating to time, place and situation
	EA	29			
Jones et al.29	GA	72	Elective hip or knee replacement	Mortality, cognitive dysfunction	Patients over the age of 60 years
	SA	74			
Juelsgaard et al.30	GA SA	14 15	Hip fracture surgery	Mortality, myocardial infarction, length of hospitalization	The authors quote: "Patients with known coronary artery disease scheduled for osteosynthesis of a femoral neck fracture were included in the study". The exclusion criteria were: uncooperative patients, recent myocardial infarction, unstable angina pectoris, significant aortic stenosis and other established contraindications for spinal anesthesia
McKenzie et al.34	GA	51	Femoral neck fracture surgery	Mortality, myocardial infarction	Patients over the age of 65 years
	SA	49			
McLaren et al. 35	GA	29	Femoral neck fracture surgery	Mortality	More patients in the spinal anesthesia group had respiratory problems
	SA	26			
Nielson et al. 37	GA	39	Elective knee arthroplasty	Cognitive dysfunction	Patients between 60 and 86 years of age
	SA	25			
Racle et al. 38	GA	35	Hip surgery	Mortality, myocardial infarction, stroke, cognitive dysfunction	The patients were women
	SA	35			
Riis et al. 39	GA	10	Total hip replacement arthroplasty	Cognitive dysfunction	The authors reported data from a third group with GA and EA combined
	EA	10			
Valentin et al.41	GA		Hip fracture surgery	Mortality	Premedication was not given to high-risk patients; there were more high-risk patients in the spinal anesthesia group
	SA				
White et al.42	GA	20	Femoral neck fracture surgery	Mortality, cognitive dysfunction, pneumonia	Patients in the spinal anesthesia group received general anesthetic drugs reported as "light anesthesia"
	SA	19			
Williams-Russo et al.44	GA	134	Total knee replacement	Mortality, length of hospitalization, cognitive dysfunction	The authors developed their own questionnaire to evaluate cognitive dysfunction
	EA	128			

**Figure 4:**
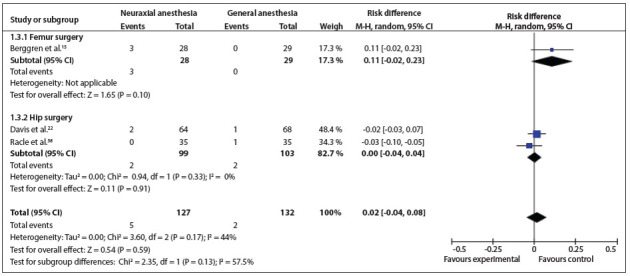
Forest plot including the three studies that analyzed stroke.

*Mortality*: This outcome was analyzed in 11 studies.^16^ ^,^ ^22^ ^,^ ^23^ ^,^ ^27^ ^,^ ^30^ ^,^ ^34^ ^,^ ^35^ ^,^ ^38^ ^,^ ^41^ ^,^ ^42^ ^,^ ^44^ Three types of surgery were conducted in the studies included: hip, femur and knee surgery. There were no statistically significant differences between the types of surgeries (RD = -0.01; 95% CI: -0.04 to 0.01; P = 0.25; 1903 participants) (Figure 4).^15^ ^,^ ^22^ ^,^ ^38^

**Figure 5:**
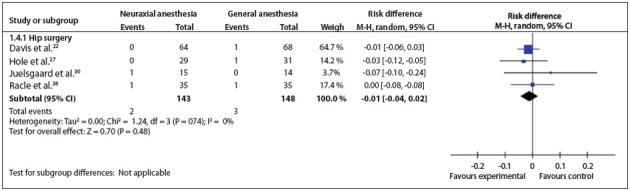
Forest plot including the four studies that analyzed myocardial infarction.

*Stroke*: This was analyzed in three studies.^15^ ^,^ ^22^ ^,^ ^38^ Two types of surgery were conducted: femur and hip surgery. There was no statistically significant difference between the types of surgery (RD = 0.02; 95% CI: -0.04 to 0.08; P = 0.17; 259 participants) (Figure 5).^15^ ^,^ ^22^ ^,^ ^38^

**Figure 6:**
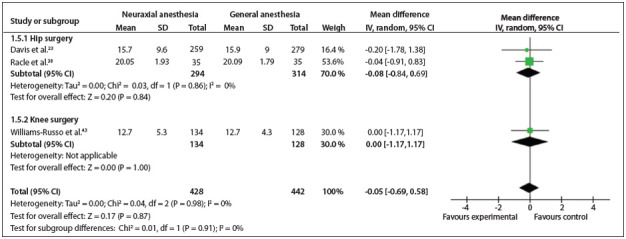
Forest plot including the three studies that analyzed length of hospital stay.

*Myocardial infarction*: Four studies analyzed this outcome.^22^ ^,^ ^27^ ^,^ ^30^ ^,^ ^38^ The outcome was analyzed only for patients who underwent hip surgery. There was no statistically significant difference (RD = -0.01; 95% CI: -0.04 to 0.02; P = 0.48; 291 participants) (Figure 6).^23^ ^,^ ^38^ ^,^ ^43^

**Figure 7:**
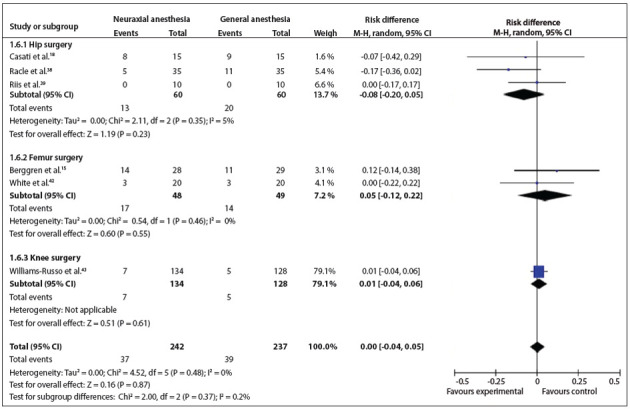
Forest plot including the six studies that analyzed postoperative cognitive dysfunction.

*Length of hospitalization*: This was analyzed in three studies.^23^ ^,^ ^38^ ^,^ ^44^ Two types of surgery were conducted: hip and knee surgeries. There was no statistically significant difference considering all types of surgeries (MD = - 0.05; 95% CI: - 0.69 to 0.58; P = 0.87; 870 participants) (Figure 7).^15^ ^,^ ^18^ ^,^ ^38^ ^,^ ^39^ ^,^ ^42^ ^,^ ^43^

**Figure 8:**
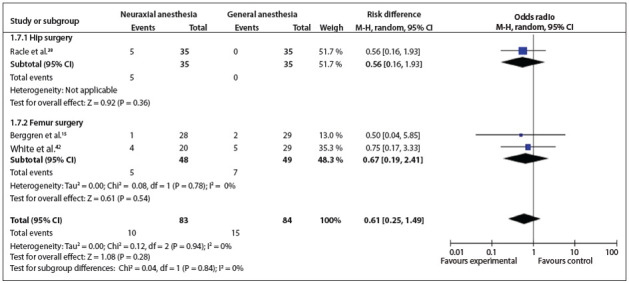
Forest plot including the three studies that analyzed pneumonia.

*Postoperative cognitive dysfunction*: this outcome was analyzed in six studies.^15^ ^,^ ^18^ ^,^ ^38^ ^,^ ^39^ ^,^ ^42^ ^,^ ^44^ Three types of surgery were conducted in the studies included. There were no statistically significant differences between the types of surgery (RD = 0.00; 95% CI: -0.04 to 0.05; P = 0.87; 479 participants) (Figure 8).^15^ ^,^ ^38^ ^,^ ^42^ ^,^ ^43^ Different authors reported using different types of instruments to analyze this outcome.

**Figure 9:**
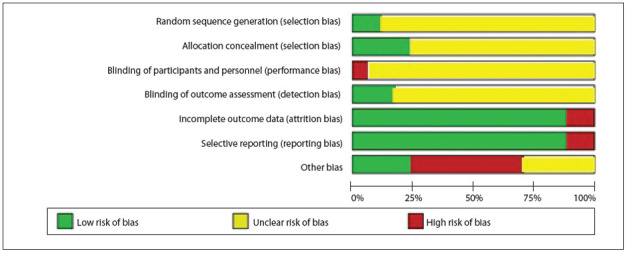
Risk of bias in studies included.

*Pneumonia*: This was analyzed in three studies.^15^ ^,^ ^38^ ^,^ ^42^ Two types of surgery were conducted: hip and femur surgery. There was no statistically significant difference between the types of surgery (OR = 0.61; 95% CI: 0.25 to 1.49; P = 0.28; 167 participants) (Figure 9).

### Sensitivity analysis

None of the authors reported dropouts but they did report the frequencies with which the outcomes occurred. The results did not change when we analyzed only the studies with the same length of follow-up time. 

One study was responsible for statistical heterogeneity in the mortality analysis.35 However, there was no statistically significant difference when the data of this study was not taken into consideration (RD = -0.01; 95% CI: -0.02 to 0.01; P = 0.45). 

The analysis on stroke showed statistical heterogeneity in a test for subgroup differences. The study by Berggren et al.^15^ was responsible for this, because more patients in the neuraxial anesthesia group presented hypotension than was seen in the general anesthesia group, and four patients were unresponsive to treatment.

## DISCUSSION

Some clinicians believe that neuraxial anesthesia is more effective and safer than general anesthesia, based on their own clinical practice. However, this systematic review was unable to prove that neuraxial anesthesia has any advantages over general anesthesia for orthopedic surgery. 

According to the Cochrane Collaboration's tool for assessing the risk of bias, the majority of the studies were generally of poor quality. Random sequence generation, allocation concealment and blinding were not described correctly, were omitted or were not conducted appropriately. The results from the studies included may therefore be limited. Appropriate reporting of the methodological items for designing and conducting studies is important for ensuring quality in a systematic review.^9^ This systematic review can be considered to present good current evidence because we pooled data from studies with the same surgical procedures, analyzed methodological flaws in the studies included before pooling the results and explored occurrences of heterogeneity. The poor quality of the studies included may place limitations on our results.

Mortality has been analyzed in other systematic reviews, without finding any statistical differences between the groups.^2^ ^,^ ^4^ ^-^ ^6^ ^,^ ^8^ We demonstrated the same result as in these previous studies, but the length of follow-up among our included studies reached six months in only one study and was less than three months in the others.

Stroke was analyzed in three RCTs, with no differences between the groups.^15^ ^,^ ^22^ ^,^ ^38^ These were small studies, and our stroke rate was 2.7% (7/259), with no statistically significant difference between the groups. Although meta-analysis can improve statistical power, our sample size was small. In this systematic review, there were no subgroup analyses. There was an analysis on the types of surgery, and this was done in all the meta-analyses. The test for the difference between the groups in the stroke analysis showed an I^2^ test result of 57.5%. We reviewed the articles included in this analysis again and noted that there was an event in the study by Berggren et al.^15^ that was not seen in any other study. These authors reported cases with arterial hypotension that did not resolve with the treatment used, which may have been responsible for the greater frequency of stroke in the neuraxial anesthesia group. On the other hand, they did not report whether this event was present in the other group, how the treatment was performed or whether the habitual treatment used in clinical practice was used in these cases.

The cardiac protective effect from neuraxial anesthesia seems to be a matter of controversy in noncardiac surgery.^45^ Parker et al. analyzed patients who underwent hip fracture surgery and found that the rate of myocardial infarction was 1% (5/505) in the neuraxial anesthesia group.^8^ Our rate for this outcome was 1.7% (5/291) with no statistically significant difference, but those authors analyzed thoracic epidural anesthesia.

Length of hospitalization was reported in three studies.^23^ ^,^ ^38^ ^,^ ^44^ Macfarlane et al. reported that neuraxial anesthesia produced a beneficial effect for total knee arthroplasty.^5^ They concluded that neuraxial anesthesia can facilitate rehabilitation and can reduce hospital stay. However, we did not observe this result.

Postoperative cognitive dysfunction was analyzed in six studies.^15^ ^,^ ^18^ ^,^ ^38^ ^,^ ^39^ ^,^ ^42^ ^,^ ^44^ Each study included used a different analysis method: Organic Brain Syndrome Scale,15 Mini Mental State Examination,18 mental changes,^38^ psychologist and attention test,^39^ mental confusion^42^ and neurophysiological tests.^44^ The results relating to this outcome are questionable.

Pneumonia was reported in three studies.^15^ ^,^ ^38^ ^,^ ^42^ Furthermore, a previous systematic review showed that this outcome was less common following neuraxial anesthesia than following general anesthesia (OR 0.37; 95% CI: 0.15 to 0.89). However, the scenario analyzed was vascular surgery, and one study in that review reported more events in the general anesthesia group than were seen in other studies.^46^ In that study, there were more smokers in the general anesthesia group and bias may have occurred. Our results were unable to prove the same effect from neuraxial anesthesia in relation to orthopedic surgery.

For future research, attention needs to be paid to the way in which random sequence generation, allocation concealment and blinding are reported. Appropriate use of these methodological criteria can improve the quality of systematic reviews.^5^ These topics need to be reported in sufficient detail for readers to be able to judge whether the results are good enough to be reproducible in clinical practice. The mortality rate can help to elucidate the effectiveness and safety of neuraxial anesthesia in comparison with general anesthesia. Making the assumptions of 5% mortality in the general anesthesia group, 1% mortality in the neuraxial anesthesia group, 80% power and 5% significance level, it will be necessary to have 284 participants in each group for future studies that analyze mortality. More RCTs with adequate numbers of patients and external and internal validity are needed. 

The implications for clinical practice are that, so far, it is not possible to say whether neuraxial anesthesia is more effective and safer than general anesthesia for orthopedic surgery. Each patient should be analyzed individually, and anesthesiologists should take into account their own previous experiences and hospital working conditions.

## CONCLUSION

So far, the evidence available from the studies included is insufficient to prove that neuraxial anesthesia is more effective and safer than general anesthesia for orthopedic surgery. However, this systematic review does not rule out clinically important differences with regard to mortality, stroke, myocardial infarction, length of hospitalization, postoperative cognitive dysfunction or pneumonia. 
